# Supplementary material for: Phytogenics and encapsulated sodium butyrate can replace antibiotics as growth promoters for lightly weaned piglets
Source: PLoS One. 2022 Dec 22;17(12):e0279197. doi: 10.1371/journal.pone.0279197 (PMC9778559; doi:10.1371/journal.pone.0279197)
Supplement: S1 File — (DOCX) [file pone.0279197.s001.docx]

**RELATÓRIO DIÁRIO NUTRIAD**

**17/01/2019**

Início do protocolo.

**18/01/2019**

Baia 36, animal 946, diarreia, 0.5 ml de corta curso.

Baia 10, animal 864, diarreia, 0.5 ml de corta curso.

Baia 7, animal 898, diarreia, 0.5 ml de corta curso.

Baia 27, animal 967, diarreia, 0.5 ml de corta curso.

Baia 19, animal 874, diarreia, 0.5 ml de corta curso.

Baia 19, animal 874, diarreia, 0.5 ml de corta curso.

Baia 23, animal 986, diarreia, 0.5 ml de corta curso.

**19/01/2019**

Baia 29, animal 957, diarreia, 0.5 ml de corta curso.

Baia 31, animal 965, diarreia, 0.5 ml de corta curso.

Baia 19, animal 791, diarreia, 0.5 ml de corta curso.

Baia 21, animal 841, diarreia, 0.5 ml de corta curso.

Baia 22, animal 970, diarreia, 0.5 ml de corta curso.

Baia 22, animal 825, diarreia, 0.5 ml de corta curso.

Baia 23, animal 986, diarreia, 0.5 ml de corta curso.

Baia 39, animal 781, diarreia, 0.5 ml de corta curso.

Baia 36, animal 809, diarreia, 0.5 ml de corta curso.

Baia 34, animal 776, diarreia, 0.5 ml de corta curso.

Baia 19, animal 895, diarreia, 0.5 ml de corta curso.

Baia 3, animal 779, diarreia, 0.5 ml de corta curso.

Baia 2, animal 973, diarreia, 0.5 ml de corta curso.

Baia 24, animal 760, diarreia, 0.5 ml de corta curso.

Baia 28, animal 911, diarreia, 0.5 ml de corta curso.

Baia 34, animal 871, diarreia, 0.5 ml de corta curso.

Baia 33, animal 780, diarreia, 0.5 ml de corta curso.

Baia 32, animal 861, diarreia, 0.5 ml de corta curso.

Baia 17, animal 831, diarreia, 0.5 ml de corta curso.

**20/01/2019**

Baia 40, animal 931, diarreia, 0.5 ml de corta curso.

Baia 29, animal 839, diarreia, 0.5 ml de corta curso.

Baia 31, animal 965, diarreia, 0.5 ml de corta curso.

Baia 29, animal 957, diarreia, 0.5 ml de corta curso.

Baia 31, animal 914, diarreia, 0.5 ml de corta curso.

Baia 30, animal 915, diarreia, 0.5 ml de corta curso.

Baia 30, animal 955, diarreia, 0.5 ml de corta curso.

Baia 32, animal 962, diarreia, 0.5 ml de corta curso.

Baia 33, animal 780, diarreia, 0.5 ml de corta curso.

Baia 36, animal 809, diarreia, 0.5 ml de corta curso.

Baia 39, animal 781, diarreia, 0.5 ml de corta curso.

Baia 39, animal 884, diarreia, 0.5 ml de corta curso.

Baia 39, animal 804, diarreia, 0.5 ml de corta curso.

Baia 28, animal 916, diarreia, 0.5 ml de corta curso.

Baia 27, animal 967, diarreia, 0.5 ml de corta curso.

Baia 27, animal 941, diarreia, 0.5 ml de corta curso.

Baia 25, animal 835, diarreia, 0.5 ml de corta curso.

Baia 24, animal 760, diarreia, 0.5 ml de corta curso.

Baia 24, animal 793, diarreia, 0.5 ml de corta curso.

Baia 25, animal 934, diarreia, 0.5 ml de corta curso.

Baia 22, animal 820, diarreia, 0.5 ml de corta curso.

Baia 22, animal 825, diarreia, 0.5 ml de corta curso.

Baia 22, animal 970, diarreia, 0.5 ml de corta curso.

Baia 21, animal 841, diarreia, 0.5 ml de corta curso.

Baia 21, animal 854, diarreia, 0.5 ml de corta curso.

Baia 21, animal 969, diarreia, 0.5 ml de corta curso.

Baia 17, animal 980, diarreia, 0.5 ml de corta curso.

Baia 5, animal 812, tosse, 0.5 ml de florfenicol.

Baia 6, animal 888, diarreia, 0.5 ml de corta curso.

Baia 7, animal 938, diarreia, 0.5 ml de corta curso.

Baia 8, animal 920, diarreia, 0.5 ml de corta curso.

Baia 9, animal 899, diarreia, 0.5 ml de corta curso.

Baia 13, animal 843, tosse, 0.5 ml de florfenicol.

Baia 13, animal 843, diarreia, 0.5 ml de corta curso.

Baia 9, animal 899, tosse, 0.5 ml de florfenicol.

Baia 9, animal 764, tosse, 0.5 ml de florfenicol.

Baia 14, animal 819, diarreia, 0.5 ml de corta curso.

Baia 37, animal 827, diarreia, 0.5 ml de corta curso.

Baia 37, animal 936, diarreia, 0.5 ml de corta curso.

Baia 18, animal 891, diarreia, 0.5 ml de corta curso.

Baia 18, animal 894, diarreia, 0.5 ml de corta curso.

Baia 23, animal 885, diarreia, 0.5 ml de corta curso.

Baia 23, animal 789, diarreia, 0.5 ml de corta curso.

Baia 34, animal 776, diarreia, 0.5 ml de corta curso.

Baia 10, animal 772, diarreia, 0.5 ml de corta curso.

Baia 10, animal 864, diarreia, 0.5 ml de corta curso.

Baia 11, animal 759, diarreia, 0.5 ml de corta curso.

Baia 24, animal 995, diarreia, 0.5 ml de corta curso.

Baia 16, animal 937, diarreia, 0.5 ml de corta curso.

Baia 16, animal 790, diarreia, 0.5 ml de corta curso.

Baia 33, animal 964, diarreia, 0.5 ml de corta curso.

Baia 29, animal 878, diarreia, 0.5 ml de corta curso.

Baia 19, animal 791, diarreia, 0.5 ml de corta curso.

Baia 26, animal 992, diarreia, 0.5 ml de corta curso.

Baia 26, animal 903, diarreia, 0.5 ml de corta curso.

**21/01/2019**

Baia 1, animal 808, diarreia, 0.5 ml de corta curso.

Baia 1, animal 875, diarreia, 0.5 ml de corta curso.

Baia 3, animal 779, diarreia, 0.5 ml de corta curso.

Baia 5, animal 982, diarreia, 0.5 ml de corta curso.

Baia 5, animal 869, diarreia, 0.5 ml de corta curso.

Baia 24, animal 760, diarreia, 0.5 ml de kinetomax.

Baia 24, animal 995, diarreia, 0.5 ml de kinetomax.

Baia 24, animal 793, diarreia, 0.5 ml de kinetomax.

Baia 25, animal 934, diarreia, 0.5 ml de kinetomax.

Baia 28, animal 911, diarreia, 0.5 ml de kinetomax.

Baia 28, animal 916, diarreia, 0.5 ml de kinetomax.

Baia 40, animal 996, diarreia, 0.5 ml de corta curso.

Baia 39, animal 884, diarreia, 0.5 ml de kinetomax.

Baia 38, animal 829, diarreia, 0.5 ml de corta curso.

Baia 37, animal 827, diarreia, 0.5 ml de corta curso.

Baia 35, animal 828, diarreia, 0.5 ml de corta curso.

Baia 37, animal 936, diarreia, 0.5 ml de kinetomax.

Baia 32, animal 861, diarreia, 0.5 ml de kinetomax.

Baia 32, animal 862, diarreia, 0.5 ml de kinetomax.

Baia 31, animal 830, diarreia, 0.5 ml de corta curso.

Baia 30, animal 955, diarreia, 0.5 ml de kinetomax.

Baia 25, animal 925, diarreia, 0.5 ml de corta curso.

Baia 8, animal 920, diarreia, 0.5 ml de corta curso.

Baia 11, animal 985, diarreia, 0.5 ml de corta curso.

Baia 14, animal 823, diarreia, 0.5 ml de corta curso.

Baia 14, animal 959, diarreia, 0.5 ml de corta curso.

Baia 15, animal 952, diarreia, 0.5 ml de corta curso.

Baia 16, animal 937, diarreia, 0.5 ml de corta curso.

Baia 18, animal 894, diarreia, 0.5 ml de kinetomax.

Baia 18, animal 891, diarreia, 0.5 ml de kinetomax.

Baia 19, animal 895, diarreia, 0.5 ml de corta curso.

Baia 19, animal 791, diarreia, 0.5 ml de kinetomax.

Baia 20, animal 922, diarreia, 0.5 ml de corta curso.

Baia 20, animal 925, diarreia, 0.5 ml de corta curso.

Baia 21, animal 824, diarreia, 0.5 ml de kinetomax.

Baia 21, animal 840, diarreia, 0.5 ml de kinetomax.

Baia 22, animal 820, diarreia, 0.5 ml de kinetomax.

Baia 22, animal 970, diarreia, 0.5 ml de kinetomax.

Baia 22, animal 825, diarreia, 0.5 ml de kinetomax.

Baia 28, animal 994, diarreia, 0.5 ml de corta curso.

Baia 3, animal 919, diarreia, 0.5 ml de corta curso.

Baia 4, animal 833, diarreia, 0.5 ml de corta curso.

Baia 5, animal 826, diarreia, 0.5 ml de corta curso.

Baia 11, animal 759, diarreia, 0.5 ml de corta curso.

Baia 14, animal 819, diarreia, 0.5 ml de corta curso.

Baia 2, animal 973, diarreia, 0.5 ml de corta curso.

Baia 1, animal 987, diarreia, 0.5 ml de corta curso.

Baia 18, animal 988, diarreia, 0.5 ml de corta curso.

Baia 19, animal 971, diarreia, 0.5 ml de corta curso.

Baia 22, animal 775, diarreia, 0.5 ml de corta curso.

Baia 36, animal 910, diarreia, 0.5 ml de corta curso.

Baia 34, animal 818, diarreia, 0.5 ml de corta curso.

**22/01/2019**

Baia 2, animal 940, diarreia, 0.5 ml de corta curso.

Baia 3, animal 919, diarreia, 0.5 ml de kinetomax.

Baia 3, animal 779, diarreia, 0.5 ml de corta curso.

Baia 4, animal 954, diarreia, 0.5 ml de corta curso.

Baia 5, animal 826, diarreia, 0.5 ml de kinetomax.

Baia 5, animal 869, diarreia, 0.5 ml de kinetomax.

Baia 6, animal 956, diarreia, 0.5 ml de corta curso.

Baia 8, animal 981, diarreia, 0.5 ml de corta curso.

Baia 11, animal 759, diarreia, 0.5 ml de corta curso.

Baia 14, animal 823, diarreia, 0.5 ml de kinetomax.

Baia 16, animal 993, diarreia, 0.5 ml de corta curso.

Baia 19, animal 791, diarreia, 0.5 ml de kinetomax.

Baia 19, animal 895, diarreia, 0.5 ml de kinetomax.

Baia 22, animal 970, diarreia, 0.5 ml de corta curso.

Baia 22, animal 825, diarreia, 0.5 ml de corta curso.

Baia 22, animal 858, diarreia, 0.5 ml de corta curso.

Baia 22, animal 820, diarreia, 0.5 ml de corta curso.

Baia 25, animal 934, diarreia, 0.5 ml de corta curso.

Baia 27, animal 967, diarreia, 0.5 ml de corta curso.

Baia 28, animal 994, diarreia, 0.5 ml de corta curso.

Baia 39, animal 755, diarreia, 0.5 ml de corta curso.

Baia 38, animal 966, diarreia, 0.5 ml de corta curso.

Baia 37, animal 936, diarreia, 0.5 ml de corta curso.

Baia 35, animal 828, diarreia, 0.5 ml de kinetomax.

Baia 35, animal 761, diarreia, 0.5 ml de corta curso.

Baia 34, animal 768, diarreia, 0.5 ml de corta curso.

Baia 33, animal 913, diarreia, 0.5 ml de corta curso.

Baia 32, animal 861, diarreia, 0.5 ml de corta curso.

Baia 29, animal 957, diarreia, 0.5 ml de corta curso.

**23/01/2019**

Início da medicação via água com Amoxicilina e Tiamulina, durante 7 dias.

Baia 14, animal 823, diarreia, 0.5 ml de corta curso.

Baia 25, animal 835, diarreia, 0.5 ml de corta curso.

Baia 4, animal 954, diarreia, 0.5 ml de corta curso.

Baia 3, animal 766, tosse, 0.5 ml de florfenicol.

Baia 26, animal 860, diarreia, 0.5 ml de corta curso.

**24/01/2019**

Baia 29, animal 957, diarreia, 0.5 ml de corta curso.

Baia 37, animal 828, diarreia, 0.5 ml de corta curso.

Baia 14, animal 959, diarreia, 0.5 ml de corta curso.

Baia 23, animal 885, encefalite, 0.5 ml de amoxicilina e 0.1 de diclofenaco.

Pesagem de todos os animais individualmente.

Baia 23, animal 892 morreu (morte súbita)

Peso animal 892: 6,515 kg

Peso baia: 23,925 (4 animais)

Ração fornecida: 15 kg

Ração consumida: 4,725 kg

**26/01/2019**

Baia 10, animal 774, diarreia, 0.5 ml de corta curso.

Baia 40, animal 931, diarreia, 0.5 ml de corta curso.

Baia 24, animal 870, encefalite, 0.5 ml de amoxicilina e 0.1 ml de diclofenaco.

**28/01/2019**

Baia 5, animal 869, diarreia, 1 ml de corta curso.

Baia 5, animal 812, tosse, 0.5 ml de florfenicol.

Baia 24, animal 870, encefalite, 0.5 ml de amoxicilina.

Baia 34, animal 983 morreu (morte súbita)

Peso animal 983: 6,08 kg

Peso baia: 22,35 kg (4 animais)

Ração fornecida: 15 kg

Ração consumida: 9,955 kg

**29/01/2019**

Baia 6, animal 888, diarreia, 1 ml de corta curso.

Baia 5, animal 812, tosse, 0.5 ml de florfenicol.

Baia 20, animal 877, problema de pele, 0.5 ml de amoxicilina e banho de iodo.

Baia 20, animal 922, problema de pele, 0.5 ml de amoxicilina e banho de iodo.

Baia 24, animal 870 retirado do protocolo (encefalite)

Peso animal 870: 4,445 kg

Peso baia: 20,74 (4 animais)

Ração fornecida: 15 kg

Ração consumida: 11,67 kg

**30/01/2019**

Baia 6, animal 888, diarreia, 1 ml de corta curso.

Baia 6, animal 832, diarreia, 1 ml de corta curso.

Baia 35, animal 828, diarreia, 1 ml de corta curso.

Vacinação de todos os animais com Glasser, PCV-m e para Streptococcus.

**31/01/2019**

Baia 24, animal 793, diarreia, 1 ml de corta curso.

Baia 6, animal 832, diarreia, 1 ml de corta curso.

Baia 7, animal 956, diarreia, 0.5 ml de corta curso.

Baia 10, animal 774, diarreia, 0.5 ml de corta curso.

Baia 35, animal 828, diarreia, 0.5 ml de corta curso.

Pesagem de todos os animais individualmente.

Final da ração pré inicial 1, início da ração pré inicial 2.

**01/02/2019**

Baia 10, animal 774, diarreia, 0.5 ml de corta curso.

Baia 23, animal 789, diarreia, 1 ml de corta curso.

Baia 35, animal 828, diarreia, 1 ml de corta curso.

Baia 34, animal 768, diarreia, 1 ml de corta curso.

Baia 6, animal 832, diarreia, 0.5 ml de kinetomax.

**02/02/2019**

Baia 18, animal 988, diarreia, 1 ml de corta curso.

Baia 18, animal 988, pneumonia, 0.5 ml de florfenicol e 0.5 ml de diclofenaco.

Baia 16, animal 845, tosse, 0.5 ml de florfenicol.

Baia 21, animal 840, diarreia, 0.5 ml de corta curso.

Baia 33, animal 882, diarreia, 1 ml de corta curso.

Baia 14, animal 821, fraco, 0.5 ml de florfenicol.

Baia 29, animal 857, diarreia, 1 ml de corta curso.

**04/02/2019**

Baia 1, animal 875, dermatite, 0.5 ml de amoxicilina e banho de iodo.

Baia 5, animal 812, fraco, 0.5 ml de florfenicol.

Baia 14, animal 821, fraco, 0.5 ml de florfenicol.

Baia 27, animal 754, fraco, 0.5 ml de florfenicol.

Baia 39, animal 804, fraco, 0.5 ml de florfenicol.

Baia 20, animal 990, fraco, 0.5 ml de florfenicol.

Baia 18, animal 988, pneumonia, 0.5 ml de florfenicol.

Baia 16, animal 993, pneumonia, 0.5 ml de florfenicol.

Baia 16, animal 845, fraco, 0.5 ml de florfenicol.

**05/02/2019**

Baia 18, animal 988, encefalite, 0.5 ml de amoxicilina e 0.5 ml de diclofenaco.

Baia 24, animal 793, fraco, 0.5 ml de florfenicol.

Baia 24, animal 760, fraco, 0.5 ml de florfenicol.

Baia 24, animal 934, fraco, 0.5 ml de florfenicol.

Baia 28, animal 911, dermatite, 0.5 ml de amoxicila e banho de iodo.

Baia 33, animal 882, diarreia, 1 ml de corta curso.

**06/02/2019**

Baia 18, animal 988, encefalite, 0.5 ml de kinetomax.

Baia 17, animal 980, tosse, 0.5 ml de kinetomax.

Baia 19, animal 901, tosse, 0.5 ml de kinetomax.

Baia 20, animal 925, diarreia, 1 ml de corta curso.

Baia 25, animal 950, tosse, 0.5 ml de kinetomax.

Baia 36, animal 910, tosse, 0.5 ml de kinetomax.

Baia 36, animal 946, diarreia, 1 ml de corta curso.

Baia 33, animal 882, diarreia, 1 ml de corta curso.

Baia 5, animal 982, tosse, 0.5 ml de kinetomax.

Baia 14, animal 821, tosse, 0.5 ml de kinetomax.

Baia 7, animal 938, diarreia, 1 ml de corta curso.

**07/2/2019**

Baia 15, animal 902, tosse, 0.5 ml de kinetomax.

Baia 16, animal 993, tosse, 0.5 ml de kinetomax.

Baia 20, animal 877, diarreia, 1 ml de corta curso.

Baia 20, animal 925, diarreia, 1 ml de corta curso.

Baia 20, animal 922, dermatite, 0.5 ml de amoxicilina e banho de iodo.

Baia 24, animal 760, diarreia, 1 ml de corta curso.

Baia 24, animal 793, tosse, 0.5 ml de kinetomax.

Baia 28, animal 911, dermatite, 0.5 ml de amoxicilina e banho de iodo.

Baia 39, animal 804, fraco, 0.5 ml de florfenicol.

Baia 37, animal 936, diarreia, 1 ml de corta curso.

Baia 36, animal 778, fraco, 0.5 ml de florfenicol.

Baia 35, animal 828, tosse, 0.5 ml de kinetomax.

Baia 30, animal 955, tosse, 0.5 ml de kinetomax.

Baia 1, animal 875, dermatite, 0.5 ml de amoxicilina e banho de iodo.

Baia 4, animal 833, tosse, 0.5 ml de kinetomax.

Baia 9, animal 764, pneumonia, 0.5 ml de kinetomax.

Pesagem de todos os animais individualmente.

**09/02/2019**

Baia 1, animal 875, dermatite, 0.5 ml de amoxicilina e banho de iodo.

Baia 11, animal 856, fraco, 0.5 ml de florfenicol.

Baia 11, animal 847, fraco, 0.5 ml de florfenicol.

Baia 13, animal 816, fraco, 0.5 ml de florfenicol.

Baia 28, animal 911, dermatite, 0.5 ml de amoxicilina e banho de iodo.

Baia 24, animal 793, diarreia, 1 ml de corta curso.

Baia 36, animal 778, pneumonia, 0.5 ml de kinetomax.

Baia 20, animal 842, dermatite, 0.5 ml de amoxicilina e banho de iodo.

Baia 1, animal 917, tosse, 0.5 ml de kinetomax.

**10/02/2019**

Baia 36, animal 778, diarreia, 0.5 ml de corta curso.

Baia 24, animal 793, diarreia, 0.5 ml de corta curso.

**11/02/2019**

Pesagem de todos os animais individualmente.

Final da ração pré inicial 2, início da ração inicial.

**13/02/2019**

Baia 2, animal 851, tosse, 0.5 ml de florfenicol.

**14/02/2019**

Baia 1, animal 987, diarreia, 0.5 ml de corta curso.

Baia 5, animal 826, dermatite, 0.5 ml de ceftiomax e banho de iodo.

Baia 5, animal 812, dermatite, 0.5 ml de ceftiomax e banho de iodo.

Baia 5, animal 982, dermatite, 0.5 ml de ceftiomax e banho de iodo.

Baia 6, animal 888, dermatite, 0.5 ml de ceftiomax e banho de iodo.

Baia 10, animal 772, dermatite, 0.5 ml de ceftiomax e banho de iodo.

Baia 12, animal 800, dermatite, 0.5 ml de ceftiomax e banho de iodo.

Baia 14, animal 823, dermatite, 0.5 ml de ceftiomax e banho de iodo.

Baia 25, animal 934, dermatite, 0.5 ml de ceftiomax e banho de iodo.

Baia 24, animal 995, dermatite, 0.5 ml de ceftiomax e banho de iodo.

Baia 20, animal 922, dermatite, 0.5 ml de ceftiomax e banho de iodo.

Baia 16, animal 845, dermatite, 0.5 ml de ceftiomax e banho de iodo.

Baia 29, animal 878, dermatite, 0.5 ml de ceftiomax e banho de iodo.

Baia 24, animal 995, tosse, 0.5 ml de florfenicol.

Pesagem de todos os animais individualmente.

**17/02/2019**

Baia 16, animal 993 morreu (morte súbita)

Peso animal 993: 10,85 kg

Peso baia: 43,95 (4 animais)

Ração fornecida: 30 kg

Ração consumida: 16,4 kg

**21/02/2019**

Pesagem de todos os animais individualmente.

**23/02/2019**

Baia 2, animal 853, tosse, 0.5 ml de florfenicol.

**25/02/2019**

Baia 14, animal 821, pneumonia, 0.5 ml de florfenicol e 0.5 ml de diclofenaco.

**26/02/2019**

Baia 23, animal 821 morreu (pneumonia)

Peso animal 821: 7,8 kg

Peso baia: 51,5 (4 animais)

Ração fornecida: 60 kg

Ração consumida: 49,75 kg

**27/02/2019**

Pesagem de todos os animais individualmente.

**28/02/2019**

Abate de 40 animais, sendo 1 por baia, para coleta de fezes do ceco.

Baia 3, animal 766, tosse, 0.5 ml de kinetomax.

Baia 4, animal 932, tosse, 0.5 ml de kinetomax.

Baia 9, animal 912, tosse, 0.5 ml de kinetomax.

Baia 12, animal 771, tosse, 0.5 ml de kinetomax.

**03/03/2019**

Baia 4, animal 954, tosse, 1 ml de kinetomax.

Início do tratamento via água com Amoxicilina, durante 5 dias.

**04/03/2019**

Baia 17, animal 831, tosse, 1 ml de kinetomax e 0.5 ml de diclofenaco.

Baia 19, animal 901, tosse, 1 ml de kinetomax.

Baia 21, animal 854, tosse, 1 ml de kinetomax.

Baia 26, animal 756, tosse, 1 ml de kinetomax.

Baia 40, animal 824, tosse, 1 ml de kinetomax.

Baia 7, animal 923, tosse, 1 ml de kinetomax.

Baia 2, animal 853, tosse, 1 ml de kinetomax.

**05/03/2019**

Baia 40, animal 824, tosse, 1 ml de kinetomax.

Baia 26, animal 992, tosse, 1 ml de kinetomax.

Baia 22, animal 858, tosse, 1 ml de kinetomax.

Baia 22, animal 825, tosse, 1 ml de kinetomax.

Baia 32, animal 861, tosse, 1 ml de kinetomax.

Baia 10, animal 864, tosse, 1 ml de kinetomax.

Baia 11, animal 759, tosse, 1 ml de kinetomax.

Baia 5, animal 812, tosse, 1 ml de kinetomax.

Baia 1, animal 875, tosse, 1 ml de kinetomax.

Baia 4, animal 881, tosse, 1 ml de kinetomax.

Baia 11, animal 847, tosse, 1 ml de kinetomax.

**08/03/2019**

Pesagem de todos os animais individualmente.

Encerramento do protocolo.
