# Supplementary material for: Phytogenics and encapsulated sodium butyrate can replace antibiotics as growth promoters for lightly weaned piglets
Source: PLoS One. 2022 Dec 22;17(12):e0279197. doi: 10.1371/journal.pone.0279197 (PMC9778559; doi:10.1371/journal.pone.0279197)
Supplement: Attachment — Submitted filename: Response to Reviewers.docx [file pone.0279197.s004.docx]

**Response to Reviewers**

Requirements

Reviewer #2: The work adequately describes the use of phytogenics and encapsulated sodium butyrate in the microbiome analysis of lightly weaned piglets. However, other response variables such as intestinal morphology, lesions, and body weight gain have yet to be analyzed to consider that phytogenics and encapsulated sodium butyrate can replace antibiotics as growth promoters. It is also necessary to indicate more strongly the new knowledge resulting from the work and how it differs from similar works.

Responses

- In accordance with the reviewer's requirements, we have included two texts in lines 340 to 350 and 378 to 388 that address, respectively, the points that required clarification on the effectiveness of the evaluated additives, compared to enramycin, in terms of its effect on performance and diarrhea control; and the demonstration of what is the differential of the work, that is, what makes it different in relation to other studies that have the same characteristic.

- For the first demand, we focused on the role of tested additives against growth-promoting antibiotics with a spectrum of action against gram-positive bacteria.

- To support these two demands, we included six bibliographies, which were standardized according to the journal's rules.
